# COVID-19 Mitigation Steps Provide a Blueprint for Malaria Control and Elimination

**DOI:** 10.4269/ajtmh.20-0394

**Published:** 2020-05-07

**Authors:** Manju Rahi, Payal Das, Amit Sharma

**Affiliations:** 1 Indian Council of Medical Research, New Delhi, India;; 2 ICMR–National Institute of Malaria Research, New Delhi, India;; 3 International Centre for Genetic Engineering and Biotechnology, New Delhi, India

## Abstract

Most countries around the world have responded promptly to the novel coronavirus disease (COVID-19) challenge by adopting considered and scientifically guided strategies for its containment. However, the situation is more complex for nations where malaria is endemic, as they now have the additional burden of COVID-19. In such nations, the healthcare systems are either in the preparatory or containment phase of the current pandemic. This enforced, sudden, and sharp public health refocus is likely to result in the disruption of annual malaria control activities such as distribution of insecticide-impregnated bed nets, indoor residual spraying of insecticide, maintenance of malaria surveillance, and mass provision of antimalarial drugs. Nonetheless, we feel that the best facets of COVID-19 public health management can become new guiding principles in malaria-endemic countries to improve malaria control and hasten malaria elimination. Redirection against malaria of the best public health initiatives used in COVID-19 containment could fast-track the global goal of a malaria-free world. Such public health advancement could be one positive outcome from the scourge of COVID-19.

## INTRODUCTION

### Impact of COVID-19 on healthcare systems.

The emergence and rapid spread of novel coronavirus disease (COVID-19) across the world has created massive global disruptions, and its impacts are especially worrisome for healthcare systems, social services, and economic activities within malaria-endemic countries.^1^ Although COVID-19 containment and mitigation efforts are underway, mobilizing resources can be a daunting task for countries that are resource scarce and have relatively poor healthcare systems, as is the case for most countries where malaria remains a major public health problem.^2^ Nearly half of the global population lives in malaria-endemic regions, with an estimated 228 million cases and 400,000 deaths in 2018.^3^ The ongoing malaria struggle, in the backdrop of the COVID-19 pandemic, is creating a public healthcare management dilemma. A significant added burden is the increased risk posed to malaria frontline workers from COVID-19 patients in the community, where antimalarial measures need to be implemented routinely.

### Malaria in times of COVID-19.

Whereas COVID-19 is fittingly distracting for all nations, it is essential that other killer infectious diseases such as malaria are not ignored. In the recent past, Ebola outbreaks in West Africa led to substantial increases in morbidity and mortality due to other diseases, including malaria.^1^ This lesson must be borne in mind within malaria-afflicted countries; their redirection of attention to COVID-19 should not come at the expense of efforts to control malaria.^4^ Sustained vector control and timely management of malaria cases are the backbone of malaria control and elimination strategies. Thus, COVID-19 activities can be expected to impact ongoing or planned malaria control activities at multiple levels.

The main malaria interventional tools that could experience disruptions in COVID-19 times are in^1^ deployment of insecticide-treated nets (ITNs),^2^ indoor residual spraying of insecticides in homes,^3^ delivery of larvicides,^4^ use of rapid diagnostic test and slide-based diagnostics,^5^ appropriate treatment of malaria,^6^ malaria chemoprevention programs, and^7^ routine malaria surveillance.^4^ The lockdown tactic adopted by many countries to contain COVID-19 is also impacting global health product manufacturing and supply chains, potentially derailing distribution of antimalarial drugs, diagnostic kits, insecticides, and ITNs.^5^ A recent modeling analysis by the WHO on the impact of COVID-19 on malaria control in sub-Saharan Africa predicts a > 20% rise in malaria cases and a doubling of malaria deaths, driven by 75% reduction in both routine ITN distribution and access to antimalarials. Therefore, in many malarious countries, a public health catastrophe due to resurgence of malaria may be of greater magnitude than that directly due to the COVID-19 pandemic.^6^

### Interventional tools used in COVID-19 may be used for malaria.

Despite the generally grim world health scenario, several positives are emerging from the COVID-19 crisis. Although malaria (an old scourge) and COVID-19 (a newly emergent disease) may compete for public health attention, we feel that the best facets of current COVID-19 management provide lessons that may enable faster control and elimination of malaria. These include the following.

1. Proactive political leadership: Governments in most nations have taken bold and decisive steps (like lockdowns), often in consultation with the WHO and with international experts in epidemiology and mathematical modeling. In addition, the new culture of almost daily press briefings in many countries has fostered a sense of accountability, transparency, and trust in national health systems. A similar display of focused political will, rational approaches, and constant oversight from national leadership towards malaria will boost control and elimination efforts.
2. Enhanced disease surveillance: Diagnostic laboratory capabilities have been expanded rapidly in most countries, and many new technological options are being explored to hasten diagnostics. Point-of-care immunodiagnostic tests are being developed rapidly,^7^ and this ardor should be reignited for malaria. Despite being well established, malaria control and elimination programs require stronger surveillance systems that are supported by point-of-care diagnostics that are robust, inexpensive, and adequately sensitive to detect asymptomatic and sub-patent malaria infections. The technological platforms that have been established for COVID-19 can be redirected toward solving the aforementioned bottlenecks in malaria.
3. Compliance to disease containment strategies: We have witnessed general adherence to the COVID-19 containment strategies promoted by the WHO and by leading epidemiologists. A distillation of best recommendations includes access to robust diagnostics, implementation of social distancing, and attention to personal hygiene. In a similar vein, national and international guidelines for malaria case management, chemoprevention, and vector control need to be adhered to rigorously.
4. Science and technology: Many nations have directed their research and development resources toward speedy development of new diagnostics, potential drugs and vaccines, and digital technology for surveillance. A sense of expectation has been conveyed from political leadership to the scientific workforce to fast-track solutions. Malaria, even more so in this elimination era, is in dire need of new options for diagnostics, therapeutics, vaccines, environmentally friendly vector control solutions, and other prevention methods. Establishment of transparent governmental systems that can adopt validated new malaria control tools, without prolonged bureaucratic processes, should now be considered. At this watershed moment for public health in most countries, multi-agency, deeply layered, and siloed malaria research and control programs should be integrated.
5. Strategic and tactical planning: Professional teams with subject expertise are being resourced as think tanks to devise short- and long-term plans for COVID-19 mitigation. Taking a cue from this, malaria programs need to renew synergies, avoid duplication, and embrace a centralized, multidisciplinary team approach that addresses both the routine management of malaria and plans for progress toward elimination. Thus, core teams of experts should remain involved for a protracted period of time so as to ensure consistent progress along the trajectories that will lead to malaria elimination.
6. Disease dashboards and digital technology: Almost all countries have been transparent in reporting COVID-19 cases in real time. Daily as well as cumulative case and mortality data are being updated on numerous websites, including that of the WHO. The data-rich and digitally driven COVID-19 response has provided a new benchmark for sharing disease data, interventional measures, and anonymized clinical profiles and outcomes. Comprehensive and updated COVID-19 incidence data sets are freely available via digital dashboards. We feel that this digital account of COVID-19 is a game changer in infectious disease epidemiology and should be implemented fully for malaria. For example, malaria dashboards could include data covering 1) clinical outcomes, 2) diagnostics used, 3) drug and insecticide resistance, 4) intervention measures, 5) malaria cases and deaths, 6) parasite species and prevalence, and 7) vector surveillance. Implementation of smartphone surveillance apps and establishment of digital chains of information and communication at all levels of health care will be helpful for success in malaria elimination.
7. International borders: Countries have implemented rigorous screening to map transitory populations and impose quarantines when required. In malaria, there is a persistent risk of disease importation, and thus focused screening at entry ports might be augmented, using innovative strategies such as interactive malaria networks between countries for prompt data sharing on cases and outbreaks. The movement of peoples across borders may entail transfer of drug-resistant parasites, as has been witnessed before in malaria. Thus, international border vigilance needs strengthening, especially for countries that are close to elimination but remain under threat from their neighbors.
8. National mobile populations: Migratory workers within countries have been tracked, stationed, and quarantined so as to avoid propagation of SARS-CoV-2 infection.^8^ In malaria-endemic countries, labor is often migratory, and frequent movement from villages to cities provides an avenue for migration of malaria parasites. Be it COVID-19 or established diseases like malaria, the marginalized sections of society who drive development usually lack access to healthcare services. This mobile workforce carries threat of reintroduction of malaria into previously free zones. Thus, when relevant, transitory labor may be tracked, tested, and treated for malaria.
9. Community empowerment: We have witnessed extensive information coverage of the current pandemic. This awareness can educate and empower the masses about COVID-19, its transmission and control measures. The communication lines between governments, healthcare providers, and communities have been opened up in an unprecedented manner. Encouragingly, populations have generally displayed eagerness and compliance in adopting best healthcare practices suggested by experts. However, malaria-affected communities are usually poor on literacy, health attitudes, and acceptance of governmental guidelines. Often malaria control measures like adherence to treatment or use of vector control methods are simply doled out in communities without effective communication and community engagement, leading to poor compliance. Channels for healthcare information exchange deployed for COVID-19 should be retained for malaria.
10. Disease hotspots: Identification and containment of infection clusters is being aggressively pursued for COVID-19, as this can facilitate efforts to block SARS-CoV-2 transmission. Similarly, delineating malaria transmission hotspots will help in stemming outbreaks and eliminating malaria hotspots. In this, diagnostics, seroprevalence studies, and tools of digital technology can play key roles.
11. Free testing and treatment: In many countries, public healthcare facilities have been directed to diagnose and treat COVID-19 patients at no charge. Although malaria care is often offered at no charge, additional provision of free access to private sector health care can be considered, especially in outbreak situations. This will enable stronger public–private linkages and private sector alignment with the national goals of malaria control and elimination.
12. Partnerships with industry: Businesses across the world are contributing to the fight against COVID-19. Private enterprises are now recognized as partners and stakeholders in COVID-19 mitigation. This spirit of integration between public and private businesses toward a common enemy needs to be maintained in the context of malaria. Businesses may be incentivized via tax breaks to dedicate part of their creative energy toward malaria elimination. Businesses can sponsor both research and elimination programs. Commercial viability is paramount to the private sector, and malaria does not present the best profit-making opportunities. Hence, as for COVID-19, private sector engagement with malaria control and elimination should be projected as a service to the population.
13. Reallocation of resources: In most countries, national wealth is being used in a dynamic and flexible manner for COVID-19 mitigation. The usually bureaucratic, siloed, and inflexible approaches that mark funding for medicine and science have been reshaped into conduits that display integration, fluidity, and urgency. This timely change in public health and science funding should be sustained for malaria control and elimination.
14. Self-help: Populations are being encouraged via digital technology to take preventive steps to stem SARS-CoV-2 transmission. Novel coronavirus disease–symptomatic people are being encouraged to self-report for diagnosis in centralized facilities. A positive test is followed either by home isolation or hospital admission. Although still premature for COVID-19, there is real hope of the availability of rapid diagnostic test (RDT) kits for home use to assess infection with SARS-Cov-2 and/or viral immunity. In addition, the use of masks, sanitizers, and other personal protective equipment is expanding in public life and is even mandatory in many regions. In a similar vein, empowering malaria-afflicted communities with readily available long-lasting insecticide-impregnated bed nets and RDTs can have a direct impact on malaria control, as it will empower the communities to participate in their own health care.
15. Healthcare system preparedness: In view of the COVID-19 crisis, laboratory capacities and medical care facilities have been upgraded or established in record time in many countries. This alacrity will be very useful as we approach malaria elimination. The expansion of integrated disease surveillance will potentially bring fewer malaria cases to light over time in the elimination phase, but each case will require requisite medical attention for treatment. Hence, just as during the COVID-19 pandemic, laboratory and medical facilities will need to be on high alert for speedy diagnosis and management of patients during elimination phases of malaria.

It is feasible that a dynamic and timely deployment of the intervention tools discussed here will dampen the present COVID-19 pandemic. Maximal implementation of similar tools adapted in a disease-appropriate manner should also be considered for the control and elimination of other killer diseases such as malaria.
